# Microbiome Therapeutic *Lactiplantibacillus plantarum* PMC105 for Systemic Carbapenem-Resistant *Enterobacteriaceae* Infections: Oral and Inhalation Efficacy *In Vivo*

**DOI:** 10.4014/jmb.2508.08009

**Published:** 2025-12-29

**Authors:** Faezeh Sarafraz, Hoonhee Seo, Hanieh Tajdozian, Ali Atashi, Youjin Yoon, Sukyung Kim, Ho-Yeon Song

**Affiliations:** 1 Department of Microbiology and Immunology, School of Medicine, Soonchunhyang University, Cheonan-si, Chungnam 31151, Republic of Korea; 2 K-Microbiome Institute, Soonchunhyang University, Asan-si, Chungnam 31538, Republic of Korea

**Keywords:** Carbapenem-resistant *Enterobacteriaceae*, carbapenem-resistant *Klebsiella pneumoniae*, *Lactiplantibacillus plantarum*, probiotics, mouse model

## Abstract

Carbapenem-resistant *Enterobacteriaceae* (CRE), particularly *Klebsiella pneumoniae*, are major causes of severe systemic infections due to their resistance to most antibiotics and the high associated mortality, representing a growing global health concern. In this study, we report the *in vivo* efficacy of a novel probiotic strain, *Lactiplantibacillus plantarum* PMC105, against systemic CRE infections. In a mouse model characterized by neutropenia and antibiotic-induced gut dysbiosis, infection with carbapenem-resistant *K. pneumoniae* (CRKP) resulted in 60% mortality within two weeks. However, oral administration of PMC105 significantly reduced intestinal CRKP colonization, minimized body weight loss, and resulted in 100% survival. This therapeutic effect is presumed to result from enhanced gut barrier function, driven by upregulation of the tight junction protein ZO-1 in the ileum, thereby preventing bacterial translocation and subsequent systemic dissemination. In a therapeutic model of systemic infection following translocation, intranasal administration of PMC105 reduced bacterial loads in the stool, liver, kidneys, and lungs, improved clinical symptoms, and maintained body weight, thereby increasing survival rates. Comprehensive safety evaluations, including antibiotic susceptibility testing, hemolysis, bile salt deconjugation, D-lactate production, and cytotoxicity assays, confirmed the strain's safety. These findings support the potential of PMC105 as a dual-route microbiome-based therapeutic candidate for the treatment of systemic CRE infections and warrant further clinical investigation.

## Introduction

The rise in carbapenem-resistant *Enterobacteriaceae* (CRE) has emerged as a significant global health threat, associated with high morbidity and mortality worldwide [1]. Carbapenemases, including New Delhi metallo–β–lactamase (NDM), *Klebsiella pneumoniae* carbapenemase (KPC), and OXA−48, are key contributors to the antibiotic resistance mechanisms observed in CRE [2].

*K. pneumoniae*, a Gram-negative, rod-shaped bacterium from the *Enterobacteriaceae* family, is a prominent opportunistic pathogen that critically impacts the management of patients at risk of colonization or infection by such microorganisms [3]. Its ability to cause community-acquired and hospital-acquired infections, and its tendency to develop antibiotic resistance, are critical challenges worldwide [4]. At present, carbapenem-resistant *Klebsiella pneumoniae* (CRKP) is resistant to all available antibiotics, making it extremely difficult to treat, and leaving minimal therapeutic options [5, 6]. Addressing this urgent issue requires the development of novel treatment strategies to manage these resistant bacteria and mitigate their impact on public health [7].

The Food and Agriculture Organization (FAO) and the World Health Organization (WHO) describe probiotics as “live microorganisms which, when administered in adequate amounts, confer a health benefit on the host” [8]. Probiotics can enhance gut health and immune response by producing antimicrobial substances that improve the host’s resistance to gastrointestinal infections [9, 10].

Among probiotics, *lactobacilli*, particularly *Lactiplantibacillus plantarum*, are recognized for their antimicrobial properties against various pathogens [11, 12]. *L. plantarum*, a Gram-positive bacterium, is one of the most used probiotics due to its immune-modulating and therapeutic effects [13]. Studies have demonstrated the antimicrobial impacts of *lactobacilli* against a wide range of pathogenic microorganisms, including carbapenem-resistant *Enterobacteriaceae* (CRE) [13-17], *Listeria monocytogenes* [18], *Salmonella typhimurium* [19, 20], *Escherichia coli* [21, 22], *Streptococcus mutans*, *Aggregatibacter actinomycetemcomitans* [10], *Staphylococcus aureus* [23], *Shigella* species [24], *Clostridium difficile* [25], *Pseudomonas aeruginosa* [26, 27], and *Salmonella* species [28].

This study aims to evaluate the *in vivo* antimicrobial activity of *L. plantarum*, isolated from the traditional fermented kimchi, to assess its potential to prevent and treat CRKP intestinal infections. In addition to oral delivery, an intranasal (nebulization) route was applied in this study to explore the potential of *L. plantarum* PMC105 as a systemic microbiome-based therapeutic. Although oral administration is the most common route for probiotics, intranasal delivery offers unique advantages by allowing direct contact with the nasal mucosa and respiratory epithelium, where abundant mucosa-associated lymphoid tissue (NALT) can initiate systemic immune responses. Recent experimental studies have demonstrated the feasibility of intranasal probiotic delivery; for example, strains of *L. rhamnosus* GG [29, 30], *L*. johnsonii [31], and *L. casei* [32] administered intranasally in mice reduced pathogen burden and exhibited anti-inflammatory effects. The objective is to determine whether *L. plantarum* can inhibit pathogen growth and enhance immune responses in murine models, thereby offering a novel approach to managing CRKP infections.

## Materials and Methods

### Carbapenem-Resistant *Klebsiella pneumoniae*

A clinical Carbapenem-resistant *Klebsiella* (CRKP) isolate was obtained from the Pathogenic Resource Bank at Soonchunhyang University Hospital [33]. The strain was streaked onto MacConkey agar plates (BD Difco, USA) containing 10 mg/ml imipenem (USA) and incubated for 18 h at 37°C under aerobic conditions. The selected single colonies were then cultured in MacConkey broth (BD Difco) aerobically at 37°C for 24 h. Bacterial cultures were grown to an optical density of 1.0 at 600 nm, diluted to 2 × 10^9^ CFU/ml, preserved in sterile glycerol, and stored at −80°C.

### Isolation and Preparation of Probiotics

To isolate the probiotic candidate strain, various traditional fermented foods—including kimchi, soybean products, and fermented vegetables- were used as sources. Approximately 10 g of each sample was streaked onto De Man, Rogosa, and Sharpe (MRS) agar (BD Difco) plates supplemented with vancomycin at 4 μg/ml (Sigma-Aldrich, USA) and incubated at 37°C for 18 h under a microaerophilic chamber (Daeiltech, Republic of Korea). A total of nine lactic acid bacteria isolates were initially obtained. All isolates were subjected to Gram staining and catalase testing to confirm Gram-positive, catalase-negative phenotypes. They were then screened for acid tolerance (pH 3.0), bile salt tolerance (0.3% oxgall), and antagonistic activity against CRKP using an agar well diffusion assay. Among these, three isolates produced clear inhibition zones, and one isolate demonstrated stable viability under simulated gastrointestinal conditions. This isolate showed the most potent antimicrobial activity, acid/bile tolerance, and non-hemolytic, non-cytotoxic properties. Based on these criteria, it was selected as the final probiotic candidate for further identification, characterization, and *in vivo* evaluation. The selected single colony was cultured in MRS Broth (BD Difco) and incubated at 37°C for 18 h. Spectrophotometry was used to determine the optical density of bacterial growth (OD = 1.0, 1 × 10^9^ CFU/ml), and the stocks were then prepared by adding 60% glycerol and stored at −80°C for further study. Later, the stock was cultured in Food-Grade Media (FGM broth) containing yeast-peptone standard type of F (MBcell, Republic of Korea), Tween 80 (Sigma-Aldrich), d–glucose (Sigma-Aldrich), and magnesium sulfate heptahydrate (Sigma-Aldrich) at 37°C for 24 h [14] for an *in vivo* experiment.

### Identification of the Probiotic Candidate Using 16S rRNA

After isolating the probiotic candidate, the selected strain was sent to Biofact Company in South Korea for 16S ribosomal RNA gene sequencing. The two primers used for amplification were 27f (5'-AGAGTTTGATCCTGGCTCAG-3') and 1495R (5'-CTACGGCTACCTTGTTACGA-3'), as described previously [33, 34]. PCR amplification was performed with an initial denaturation at 95°C for 3 min, followed by 30 cycles of denaturation at 95°C for 20 sec, annealing at 56°C for 40 sec, and extension at 72°C for 90 sec, with a final extension at 72°C for 5 min. The PCR products were then purified using a PCR purification kit and sequenced using the BigDye Terminator v3.1 Cycle Sequencing Kit (Thermo Fisher Scientific, USA) on an ABI PRISM 3730XL DNA Analyzer (Applied Biosystems, USA). To identify the bacterial species, the NBLAST search tool (http://www.ncbi.nlm.nih.gov/blast) was used to compare the most abundant unique sequences against the NCBI GenBank microbial database. Matches were evaluated based on their e-values and bit scores, and the taxonomy of the best-matching sequence was assigned to each query.

### Whole Genome Sequencing of *L. plantarum* via PacBio SMRT Sequencing

Whole-genome sequencing was performed by a WGS service provider (CJBioscience, Republic of Korea) to characterize our candidate probiotic. A QIAamp DNA Mini kit (Qiagen, Germany) was used to extract genomic DNA (gDNA) from the *L. plantarum* strain. Sequencing was performed using PacBio technology, with the HGAP2 protocol in PacBio SMRT Analysis 2.3.0. Circlator 1.4.0 (Sanger Institute, UK) was used to process the data, generating circularized contigs from the PacBio sequencing. Protein-coding sequences (CDSs) were estimated using Prodigal 2.6.2 [35] and categorized based on orthologous groups through the EggNOG database (http://eggnogdb.embl.de). Genes encoding tRNAs were identified using tRNAscan–SE 1.3.1 [36], while other noncoding RNAs were detected through covariance model searches in the Rfam 12.0 database [37]. The OrthoANIu algorithm-based Average Nucleotide Identity (ANI) calculator was used to compute OrthoANI values for prokaryotic genome sequences [38].

### Minimum Inhibitory Concentration (MIC) of *L. plantarum* Cell-Free Supernatant (CFS)

*L. plantarum* strain was cultured in MRS broth media (BD Difco) at 37°C for 24 h to reach maximal growth, and the turbidity was adjusted to 0.5 McFarland. The cultured media were centrifuged for 20 min at 4,000 rpm. 0.22 μm filters were used to filter the cell-free supernatant to obtain sterile CFS. An overnight culture of CRKP with a turbidity of 0.5 McFarland standard was diluted 1:100 into MacConkey broth (BD DifcoUSA) to reach a bacterial concentration of 1 × 10^6^ CFU/ml. The minimum inhibitory concentration (MIC) test was conducted using the microtiter method [39]. In brief, 100 μl of the diluted CRKP was added to the wells of a 96–well plate. A 2-fold serial dilution of CFS was prepared in MRS media, and 100 μl of each dilution was added to the wells containing the bacterial inoculum. The positive control contained only CRKP inoculum in fresh MRS medium, while the negative control contained a 1:1 mixture of MacConkey and MRS media without bacteria. The plate was then incubated at 37°C for 24 h. Bacterial growth was measured by reading the optical density at 570 nm using a microplate reader. The MIC was determined as the lowest concentration of CFS that completely inhibited visible bacterial growth.

### Time-Kill Assay

To evaluate the bactericidal activity of *L. plantarum* against CRKP, a Time-kill assay was performed using its CFS, as previously described [14, 40, 41]. The *L. plantarum* strain was cultured in MRS broth (BD Difco) and incubated overnight at 37°C under aerobic conditions. The culture was then centrifuged, and the supernatant was filter–sterilized (0.22 μm) to obtain the CFS. For the assay, 300 μl of CRKP suspension (10^8^ CFU/ml) was inoculated into 15 ml of *L. plantarum* CFS and incubated at 37°C. The Control group consisted of CRKP suspended in sterile MRS broth without CFS inoculum. Aliquots were pipetted out from each tube at predetermined time intervals, and serially diluted to determine the viable CFU of CRKP on MacConkey agar (BD Difco). The experiment was conducted in triplicate, and total surviving cells were expressed as the mean log_10_ CFU/ml. Data are presented as mean ± standard deviation (SD). Statistical significance between groups was evaluated using an unpaired Student’s *t*-test; *p* < 0.05 was considered significant.

### Preparation of *L. plantarum* Strain for *In Vivo* Administration

The *L. plantarum* strain was cultured in MRS broth (BD Difco) and incubated aerobically at 37°C for 24 h. The bacterial stock was preserved as described previously. After incubation, the culture was centrifuged at 4,000 rpm for 20 min. The supernatant was discarded, and the bacterial pellet was resuspended and adjusted to approximately 1 × 10^10^ CFU/ml for use in Model 1 and 1 × 10^8^ CFU/ml for Model 2. To determine bacterial viability, the *L. plantarum* suspension stored in saline was cultured on MRS agar (BD Difco) plates under aerobic conditions after one week, and colony counts were performed after 48 h of incubation. For *in vivo* administration, mice in the treatment group received *L. plantarum* via oral gavage in Model 1 (200 μl per mouse), while in Model 2, the probiotic was administered intranasally via nebulization (10 ml total per treatment for each mouse). Control group mice in both models received only an equivalent volume of sterile saline by oral gavage, as in the treatment groups.

### Animal Ethics Approval and Experimental Conditions

All mouse experiments in this study were conducted per the guidelines of the Institutional Animal Ethics Committee of Soonchunhyang University (Republic of Korea), under the committee’s authorization (IACUC approval number: SCH19−0053).

Nine-week-old specific pathogen-free (SPF) female BALB/c mice (Doo Yeol Biotech, Republic of Korea) were housed in individual cages and kept under standard conditions, including a room temperature (RT) of (22−24)°C with a 12 h light/dark cycle and (30−70)% humidity in an air-conditioned animal room at (23 ± 2)°C. All mice were given a one-week adjustment period prior to the experiment, and free access to food and water *ad libitum*.

The study included two experimental mouse models. In Model 1, mice were randomly assigned to three groups with n = 6 per group, whereas in Model 2, they were assigned to three groups with n = 12 per group. Model 1 was designed to assess the oral preventive effect of *L. plantarum* and its anti-infective activity during bacterial colonization, while Model 2 evaluated its nasal efficacy following infection. All experimental procedures were performed in a biosafety level 2 (BSL−2) facility at the HM−MRC animal room of Soonchunhyang University based on World Health Organization (WHO) recommendations, and the guidelines of the Ministry of Food and Drug Safety (Registration, MFDS, No. 657).

### Experimental Design to Evaluate the Potential of *L. plantarum* in CRKP-Infected Mouse Models

To evaluate the therapeutic potential of the probiotic candidate against CRKP, two infection mouse models were conducted in this research: an oral preventive model and a nasal efficacy model. In both models, mice were randomly divided into three groups: a control group, an infection group, and a treatment group. To induce neutropenia, cyclophosphamide (450 mg/kg; 200 μl/mouse, Sigma-Aldrich) was administered intraperitoneally three days (d) before infection in both models. Additionally, gut dysbiosis was induced by oral administration of vancomycin (100 mg/kg; 200 μl/mouse) on days (D) (−2 and −1). Before CRKP infection, all mice were given an oral dose of 200 μl of 0.2 M sodium bicarbonate (Sigma-Aldrich) to neutralize gastric acid and facilitate bacterial colonization. After bicarbonate administration, the mice immediately received CRKP via the same oral route. In the treatment groups, mice received approximately 1 × 10^10^ CFU/ml of *L. plantarum* via oral gavage in the preventive model and approximately 1 × 10^8^ CFU/ml intranasally via nebulization in the efficacy model. Control groups in both models received only sterile saline orally. To assess the effect of *L. plantarum* on decreasing CRKP infection, body weight changes and illness severity scores were recorded throughout the experiments. For further analysis, in Model 1, fecal samples were collected from individual mice on D (0, 2, 4, 8, and 11), and intestinal tissues were dissected on D 7. In Model 2, stool samples were collected on D (0, 2, 4, 8, and 11), while tissue samples (Lung, Kidney, and Liver) were gathered on D (0, 4, and 10). CFU per gram of samples was determined by plating each sample onto MacConkey agar containing 10 μg/ml of imipenem (~100 mg diluted in 1 ml of NaCl). In addition, Western Blot analysis was performed on intestinal tissues collected from Model 1 to determine the gut integrity. All experiments were replicated at least twice under equal conditions.

### Determining the Oral Preventive Impact of Probiotic Strain in the CRKP-Infected Mice Model

We investigated the preventive effect of *L. plantarum* against CRKP infection in the BALB/c mouse model. In this study, mice in the treatment group received *L. plantarum* at a concentration of 1 × 10^10^ CFU/ml in a 200 μl volume via oral gavage 3 d before the first CRKP infection (D (−3, −2, −1)), as well as on D (0, 2, 6, 7, and 9) post-infection. Vancomycin was administered orally for dysbiosis at a dose of 100 mg/kg (200 μl/mouse) on D (−2 and -1). Mice received 200 μl of CRKP orally at a concentration of 1 × 10^10^ CFU/mouse on D 0, following oral bicarbonate administration to neutralize gastric acid. Subsequent CRKP challenges were performed on D (2 and 7) using 1 × 10^9^ CFU/mouse. Fecal samples were collected individually from mice on D (1, 3, 5, 7, 8, and 9) post-infection to analyze bacterial colonization. Colony-forming units per gram of stool were determined by plating the samples on MacConkey agar with 10 μg/ml of imipenem (prepared by diluting ~100 mg in 1 ml of NaCl). The number of viable CRKP colonies was assessed, expressed as log_10_ CFU/g of stool. On D 7, mice from each group were randomly selected. The small intestines were cut open and preserved for further analysis. To assess the overall effect of *L. plantarum*, the mice were observed for 2 weeks following the initial infection, to monitor survival rates, illness severity scores, and body weight changes.

### Investigating the Nebulization Efficacy of the Probiotic Candidate in a CRKP-infected Mouse Model

To evaluate the therapeutic potential of *L. plantarum* via nebulization, CRKP infection was induced in mice by orally administering 200 μl of a CRKP suspension (1 × 10^9^ CFU/mouse) along with sodium bicarbonate on D (0 and 2). During the experiment, mice in the treatment group received *L. plantarum* intranasally at a concentration of 1 × 10^8^ CFU/mouse, using a nebulizer for 20 min each time. To assess treatment outcomes, biological samples, including stool and tissues, were collected at designated time points. CFU per gram was quantified by plating stool, lung, liver, and kidney samples on MacConkey agar supplemented with 10 μg/mL imipenem. These analyses were performed to evaluate bacterial load and the potential of *L. plantarum* to reduce CRKP colonization in both intestinal and systemic organs.

### Isolation of Mouse Intestinal Tissue and Protein Extraction

The integrity of the intestinal epithelium in mice was analyzed during PMC105 using a Western blot assay, following previously established protocols [42, 43]. Small intestinal tissues from mice were collected on D 7 in the preventive mouse model, flushed with phosphate-buffered saline (PBS), and cut into small fragments. Tissue pieces were homogenized in 1× RIPA lysis buffer (Abcam, UK) with a Protease Inhibitor Cocktail (Cell Signaling Technology, USA), and centrifuged at 14,000 rpm at 4 °C for 15 min. The concentration of the extracted proteins was determined using the Bicinchoninic Acid assay kit (KeyGEN BioTECH, China). After protein extraction, the samples were quantified and transferred onto polyvinylidene difluoride (PVDF) membranes. The membranes were then incubated with primary antibodies, including zonula occludens-1 (ZO−1) (Protein Tech, USA) and β–actin (Cell Signaling Technologies), followed by incubation with a horseradish peroxidase-conjugated secondary antibody (Cell Signaling Technologies). Protein bands were visualized using a ChemiDoc XRS system (Bio-Rad), and densitometric analysis was performed using ImageJ software (version 1.8.0).

### Protein Identification and Western Blot Analysis

The intestinal tissues were homogenized in a RIPA lysis buffer (Abcam, UK), supplemented with a protease inhibitor cocktail (Cell Signaling Technology), followed by centrifugation at 14,000 rpm for 15 min at 4°C. The supernatant was collected, and after protein extraction for Western blot analysis, 10 μg of protein solution was mixed with sodium dodecyl sulfate (SDS) sample buffer (KeyGEN BioTECH) and denatured by heating at 100°C for 5 min in a ThermoMixer C (Germany). Proteins were separated on a (10−20) % Tris–glycine gel (BioRad), and transferred to polyvinylidene difluoride (PVDF) membranes (Immuno–Blot PVDF, BioRad) using the TransBlot system (BioRad) for 10 min. The nonspecific binding sites of membranes were blocked using 5% nonfat milk in Tris–buffered Saline with Tween 20 (TBST) at RT for 2.5 h, followed by overnight incubation at 4°C with primary antibodies: mouse β–actin (Cell Signaling Technology) as a loading control, and ZO−1 Rabbit Polyclonal antibody (Protein Tech). Subsequently, the membranes were washed three times with TBST buffer (5 min each), and incubated with the Horseradish Peroxidase (HRP) conjugated secondary antibody (Cell Signaling Technology) for 2.5 h at RT. The membranes were again washed (5 min, 3 times), and the appropriate amount of enhanced chemiluminescence reagent was added to visualize the protein bands. The Molecular Imager ChemiDoc Imaging System (Bio-Rad) was used to detect the bands, with image analysis being conducted using Image Lab and ImageJ software.

### Acute Toxicity of Probiotic Candidate

To evaluate the oral acute toxicity of the probiotic strain, a two-week experiment was conducted using nine-week-old female BALB/c mice, following approval by the Institutional Animal Care and Use Committee (IACUC approval number: SCH25−0026). The mice were randomly divided into two groups (6 mice per group). One group received saline drinking water (control group), while the other received *L. plantarum* orally at a concentration of 1 × 10^10^ CFU/mouse (200 μl per mouse). Mice were housed individually under a 12 h light/dark cycle, with controlled temperature at (20−25)°C, and relative humidity of (30−70)%. The mice were monitored and recorded over a 14 d period for any signs of clinical symptoms, mortality, or changes in body weight. This experiment followed the guidelines outlined in the OECD Test Guideline 423, with minor modifications to assess the acute oral toxicity of *L. plantarum* in the mouse model [44].

### Safety Assessment of the Probiotic Candidate According to MFDS Guidelines

The Joint FAO/WHO Working Group introduced standardized safety criteria for probiotics in 2002 [45], which were then adopted by the Korean Ministry of Food and Drug Safety (MFDS) in 2021 [46]. To assess the safety of the probiotic candidate per these guidelines, antimicrobial susceptibility (E–test), β–hemolysis activity, bile salt deconjugation, D–lactate production, and cell cytotoxicity were evaluated.

An antibiotic strip (Liofilchem, Italy) was used to assess antibiotic susceptibility using the E–test method [47]. As advised by the European Food Safety Authority (EFSA) [48], nine antibiotics were used: ampicillin, vancomycin, gentamicin, kanamycin, streptomycin, erythromycin, clindamycin, tetracycline, and chloramphenicol. After the strips were placed in agar plates and inoculated with the strains, they were incubated for 48 h at 37°C under anaerobic conditions. The measured minimum inhibitory concentration (MIC) was compared to the MIC cut-off criteria of the European Food Safety Authority (EFSA) [49].

The hemolytic activity of the probiotic strain was assessed using blood agar plates (Kisan Bio, Republic of Korea). After the strains were streaked onto agar plates and incubated for 48 h under anaerobic conditions at 37°C, hemolytic activity was assessed based on the following observations: A small area of the medium with a discolored zone ranging from green to brown indicates alpha (α) hemolysis, which shows a reduction of hemoglobin to diffuse methemoglobin. Complete lysis of red blood cells is indicated by a clear, pale–yellow zone surrounding the colonies, which is known as beta (β) hemolysis, whereas gamma (γ) hemolysis is characterized by the absence of any visible change in the medium [50].

The bile salt deconjugation activity of the strains was assessed using a plate assay with Taurodeoxycholic acid sodium salt (TDCA, Millipore, Germany) as the substrate [51]. TDCA−MRS agar (BD Difco) plates were prepared with a sodium salt concentration of 1 mM/L. Probiotic strains were streaked onto TDCA-enriched agar plates and incubated under anaerobic conditions at 37°C for 5 d. The negative and positive controls for bile salt deconjugation were *Lacticaseibacillus rhamnosus* KCTC 5033 and *L. plantarum* KCTC 3105 (KCTC, Korea), respectively.

The D–lactate assay was used to evaluate excessive D–lactate production, which, due to the potential risk of D–lactic acidosis, is a critical safety consideration [52]. D–lactate production by the candidate probiotic strains was assessed after incubation at 37°C for 24 h in MRS broth. After incubation, the culture supernatants were collected, and D–lactate concentrations were measured using an assay kit (Abcam) according to the manufacturer’s instructions. A standard D–lactate solution was prepared for reference, and absorbance was measured using a Victor Nivo microplate reader (VICTOR Nivo, PerkinElmer Instruments, USA). *L. rhamnosus* KCTC 5033 was used as a negative control (NC) for D–lactate production.

HT29 cells were seeded at 1 × 10^4^ cells/well in a 96-well plate in DMEM (Gibco, 10313021) containing 10% FBS (Welgene, S101−07) and 1% penicillin/streptomycin (Gibco, 15140), and cultured at 37°C, 5% CO_2_ incubator for 24 h. After washing the cultured strain with PBS, several CFU ((10^7^−10^9^) CFU) of bacteria were inoculated into 1% BSA (Sigma, A7906)-washed cells using DMEM (2% FBS and no antibiotics) for 24 h. The release of lactate dehydrogenase (LDH) into the medium was measured using the cytotoxicity detection kit (Roche, 4744926001) according to the manufacturer’s protocol. *E. coli* NCCP 14538 was purchased from the National Culture Collection for Pathogens (Republic of Korea) and used as the positive control, producing toxicity. The absorbance at 490 nm was measured using a microplate reader (VICTOR Nivo, PerkinElmer Instruments) and then converted to cytotoxicity (%). All safety experiments were conducted in accordance with the probiotic safety evaluation standards of the Korean Ministry of Food and Drug Safety (KMFDS) [53].

## Results

### 16S rRNA Gene Sequence Analysis of the Probiotic Candidate

The candidate strain was identified by 16S rRNA gene sequencing, and the resulting sequences were compared with sequences in the NCBI database (Table 1). According to the analysis, the isolate’s sequence showed 99% similarity with strains of *L. plantarum*, including DSM 20174, NBRC 15891, CIP 103151, and NRRL B−14768. Furthermore, the sequence showed a high degree of similarity to other members of the *Lactobacilli*. The 16S rRNA gene sequence of the probiotic candidate generally exhibited (98−99)% sequence identity to reference sequences in the database.

### Whole-Genome Analysis Results of the Candidate Strain

**Fig. 1:**
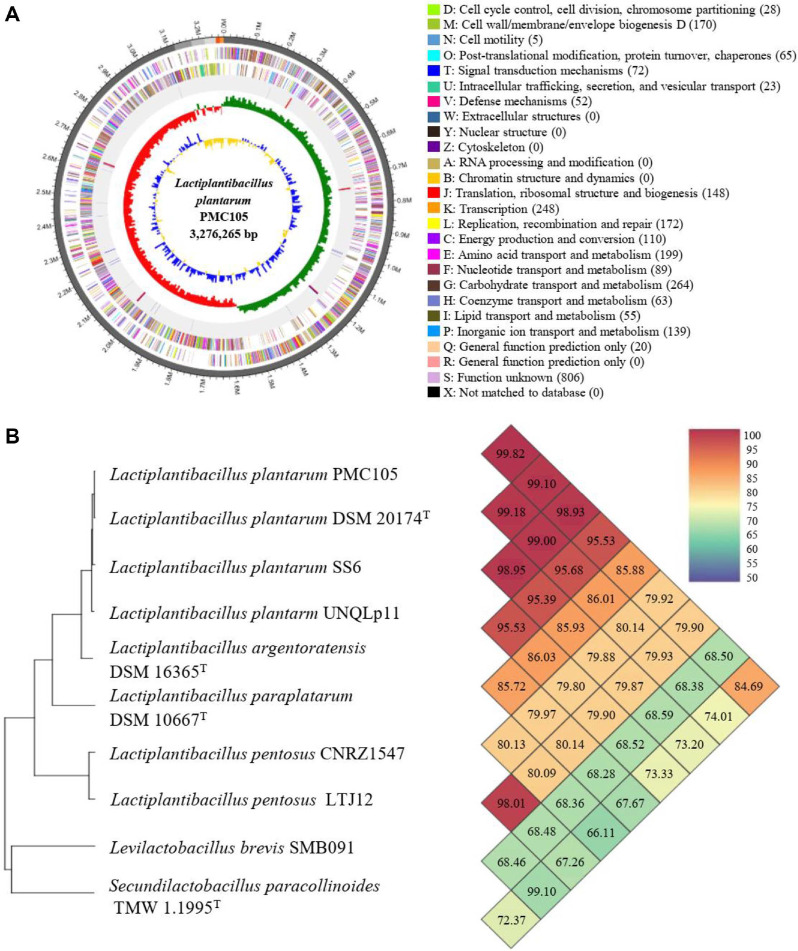
High-throughput genome sequencing of *Lactiplantibacillus plantarum* strain PMC105. (**A**) The circular map of the *L. plantarum* PMC105 strain genome shows both antisense and sense strands, colored by COG categories. RNA genes are highlighted, with tRNA in red and rRNA in blue, arranged from the outer periphery to the center. The inner circles represent the GC skew, with yellow and blue indicating positive and negative values, respectively, while the GC content is shown in red and green. This genome map was visualized using CL genomics. The relative abundance of clusters of orthologous groups (COG) functional categories of genes is presented. (**B**) Phylogenomic tree and OrthoANI result calculated with the available genomes of *Lactiplantibacillus* species. Values greater than 96% indicate that strains belong to the same species. The results for two strains are shown at the intersection of the diagonals from each strain; *e.g.*, the OrthoANI value between *L. plantarum* PMC 105 and *L. plantarum* DSM 20174 is 99.82%.

The assembled genome comprised a single circular chromosome of 3,276,265 base pairs, harboring 3,054 coding sequences (CDSs). Among these, 2,728 predicted protein-coding genes were categorized based on the functional classification of clusters of orthologous groups (COG) (Fig. 1A). Comparative genomic analysis using the Orthologous Average Nucleotide Identity (OrthoANI) approach revealed high genomic similarity with *L. plantarum*, while exhibiting lower similarity to other species within the *Lactiplantibacillus* genus, confirming its taxonomic identity as *L. plantarum* (Fig. 1B). In contrast, the *L. plantarum* isolate exhibited distinct genetic features compared to previously documented strains, supporting its designation as a novel strain, subsequently named PMC105 (Table 2).

### Determination of the Minimum Inhibitory Concentration of the Strain’s CFS

**Fig. 2:**
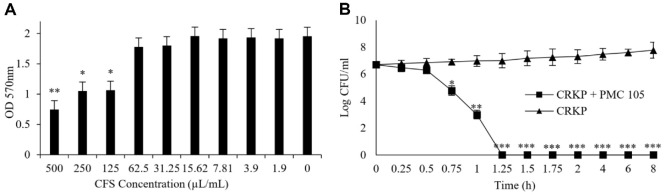
Inhibitory effect of *L. Plantarum* CFS on CRKP growth in co-culture. Antimicrobial activity of *L. plantarum* CFS against carbapenem-resistant *Klebsiella pneumoniae* (CRKP), evaluated using concentration-dependent and timedependent assays. (**A**) The minimum inhibitory concentration (MIC) of *L. plantarum* CFS against CRKP was determined by assessing bacterial growth inhibition at varying CFS concentrations. A significant reduction in CRKP growth was observed at concentrations of 125 μl/ml and above. (**B**) Time–kill assay performed with *L. plantarum* against CRKP over an 8 h period. CRKP was co-cultured with CFS of *L. plantarum*, and bacterial counts were determined by plating on MacConkey agar at each time point. *L. plantarum* CFS showed a total killing effect against CRKP after 1.25 h in co-culture. All data are expressed as the mean ± SD from triplicate experiments. Statistical significance was determined using unpaired Student’s *t*–test (***p < 0.001; **p < 0.01; *p < 0.05).

Fig. 2A shows the results of the Minimum Inhibitory Concentration (MIC). Optical density (OD) was measured at 570 nm using a microplate reader to assess bacterial growth in each well. The MIC was determined as the lowest CFS concentration from *L. plantarum* that completely inhibited the growth of CRKP. It was found to be 125 μl/ml. Subsequently, the contents of the wells that showed no growth were cultured on fresh MacConkey agar plates. These results indicate the significant antimicrobial effects of *L. plantarum* CFS against CRKP, warranting further investigation and potential therapeutic application.

### Time-kill Assay of CFS of *L. plantarum* Strain against CRKP Infection

The time-kill assay illustrated that the CFS of *L. plantarum* PMC105 showed a strong bactericidal effect against CRKP. A progressive reduction in bacterial viability was observed over time in the presence of *L. plantarum* CFCS, with complete inhibition of CRKP growth observed after 1.25 h of incubation, demonstrating the kill effect of *L. plantarum* PMC105 CFS against CRKP. On the other hand, bacterial levels in the control group (CRKP in MRS broth without CFS) were progressively increased throughout the assay. Notably, a ≥3 log_10_ CFU/ml decrease in CRKP counts was recorded for 8 h in samples treated with *L. plantarum* CFS, indicating significant time-dependent bactericidal activity. These findings illustrate that *L. plantarum* PMC105 CFS can significantly inhibit the growth and viability of CRKP under *in vitro* conditions (Fig. 2B).

### Therapeutic Impact of *L. plantarum* in a Preventive Mouse Model of CRKP

**Fig. 3:**
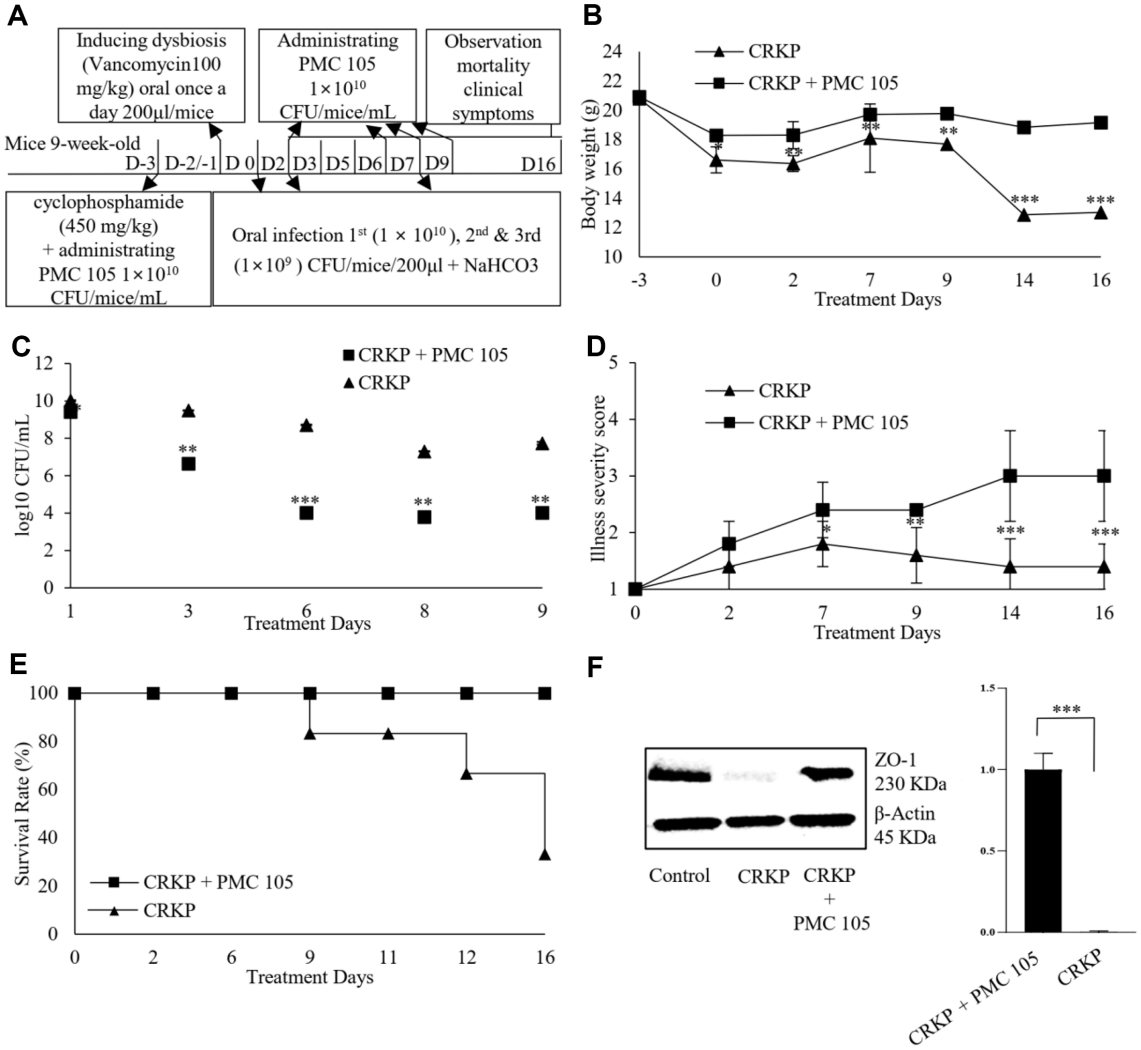
Preventive impact of probiotics in a CRKP-infected mouse model. (**A**) *L. plantarum* PMC105 was administered 3 d before infection to assess the probiotic’s preventive effect during 20 d of the experimental period. The infection was established after administering an antibiotic (vancomycin). (**B**) Body weight changes were tracked over 16 d postinfection. The untreated group showed a greater reduction in body weight than the treated group, with notable differences observed on D (14 and 16). (**C**) Stool samples were collected post-infection, and CRKP colonization was evaluated by stool CFU counts. Mice pre-treated with *L. plantarum* showed a significant reduction in CRKP viable counts throughout the study, compared to the untreated group. (**D**) The severity of illness was scored over the study using a scale of (1 to 5): (1, healthy; 2, minimally ill; 3, moderately ill; 4, severely ill; 5, dead). The untreated group showed a notable increase in illness severity, resulting in a significant rise in illness scores from D 7 onward. In contrast, the group treated with *L. plantarum* illustrated lower illness severity scores, with statistically significant differences observed from D (9 to 16). (**E**) The survival rate of mice was monitored for 2 weeks post-infection. All mice in the treated group survived throughout the experiment, whereas in the untreated group, a 66.7% mortality rate was observed by D 16. (**F**) Expression of the tight junction protein ZO−1 in small intestinal tissues was established by Western blot. *L. plantarum* pre-treatment significantly restored ZO−1 expression, compared to the untreated infected group. Each group consisted of six mice (*n* = 6). Data are presented as the mean ± SEM. Statistical significance was determined using an unpaired Student’s *t*-test (*p* < 0.05, *p < 0.01, **p < 0.001).

The study assessed the therapeutic effects of *L. plantarum* on lethal CRKP infections in a mouse model. Mice were given *L. plantarum* at a concentration of 1 × 10^10^ CFU/ml, before oral infection with CRKP (Fig. 3A). The untreated group experienced more severe weight loss compared to the treated group, with body weights significantly lower by D (14 and 16) (*p* < 0.05) (Fig. 3B). From D 2 onwards, the CRKP growth rate decreased in the group treated with *L. plantarum*. The mice were monitored for two weeks, with CFUs measured throughout. By D 14, CRKP had reduced the viable counts in the treated group (*p* < 0.01). Stool samples from the pre-treated mice showed lower CRKP colony numbers compared to those from other groups until the study’s conclusion. Consequently, significant differences in CFU counts were observed between the pre-treated group (prior to *L. plantarum* treatment) and the untreated group (Fig. 3C). After oral infection, the untreated mice exhibited symptoms such as diarrhea and fatigue. There were considerable differences in illness severity scores between the treated and untreated groups during the study (*p* < 0.001) (Fig. 3D). All mice treated with *L. plantarum* survived 14 d post-infection, whereas 66.7% of the mice infected with CRKP in the untreated group died during the study (Fig. 3E). In addition, the Western blot assay of small intestine on D 7 illustrated a noticeable reduction in ZO−1 levels in the infection group that significantly improved gut barrier integrity by reducing intestinal permeability by PMC105 treatment rather than the treatment group, which is evidenced by the high level of tight junction protein ZO−1 in the treated mice group (Fig. 3F).

### Nebulization Potential of *L. plantarum* on *Klebsiella pneumoniae* Induced in a Mouse Model

**Fig. 4:**
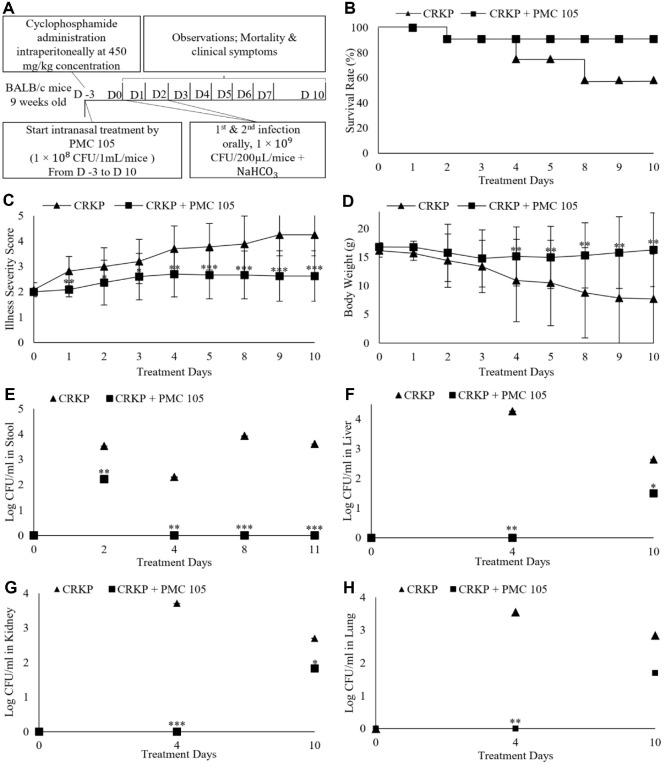
Nebulization Effect of Probiotics on CRKP-infected mice. (**A**) The impact of intranasally administered *L. plantarum* against oral CRKP-infected mice was assessed using a nebulizer over a 2-week experiment. Mice received *L. plantarum* via nebulization prior to CRKP oral challenge, followed by clinical monitoring and microbiological assessment. (**B**) Observation of mouse mortality showed that all mice treated with *L. plantarum* survived, whereas 40% of untreated mice died. (**C**) Illness severity was assessed throughout the study using a (1 to 5) scale (1, healthy; 2, minimally ill; 3, moderately ill; 4, severely ill; 5, dead). The *L. plantarum*-treated group demonstrated lower illness severity scores, with notable differences compared to the untreated group observed from D (4 to 10). In contrast, the untreated group showed a gradual increase in illness severity over the 10 d. (**D**) Body weight was monitored during the experiment. The probiotic candidate reduced body weight in the untreated group compared to the treated group. (**E−H**) To assess the decolonization effect of probiotics, stool samples and organs (including liver, kidney, and lung) were collected post-infection, and CFU assays for CRKP were performed. There was a significant reduction in CRKP viability in *L. plantarum*-treated mice. Each group consisted of six mice (*n* = 12). Statistical significance with controls was analyzed using unpaired Student’s *t*–test (***p < 0.001; **p < 0.01; *p < 0.05).

Mice were given *L. plantarum* intranasally at a concentration of 1 × 10^8^ CFU/ml for 20 min, before oral infection with CRKP (Fig. 4A). 60% of the infected mice in the untreated group with CRKP survived during the experiment. No mortality was observed in the nebulization treatment group of mice treated with *L. plantarum* (Fig. 4B). Following oral infection, untreated mice exhibited symptoms such as diarrhea and weakness. Significant differences in illness scores were observed between the treated and untreated groups from D (4 to 10) (*p* < 0.001) (Fig. 4C). The untreated group illustrated greater weight loss compared to the treated group, with their body weight significantly reduced on D (14 and 16) (*p* < 0.05) (Fig. 4D). A microbiological assessment of the normal intestinal microflora in mice was conducted by culturing fecal samples from uninfected mice on MacConkey agar plates containing 10 mg/ml imipenem. After 24 h of incubation, no bacterial growth was detected on the plates for any of the fecal samples, indicating the absence of CRE strains. The CFU of collected samples (stool, liver, kidney, and lung) was measured during the study. The treated group showed notable differences in CFU counts compared to the untreated group. CRKP reduced viable colony counts in the treated group, rather than in the untreated group, by D 10 (*p* < 0.01) (Fig. 4E−4H). The results demonstrated that although CRKP can colonize the intestines of mice, Nebulization with *L. plantarum* PMC105 reduced the bacterial burden and showed a trend toward improved survival compared with the CRKP-infected group.

### Acute Oral Toxicity of the Investigated *L. plantarum*

**Fig. 5:**
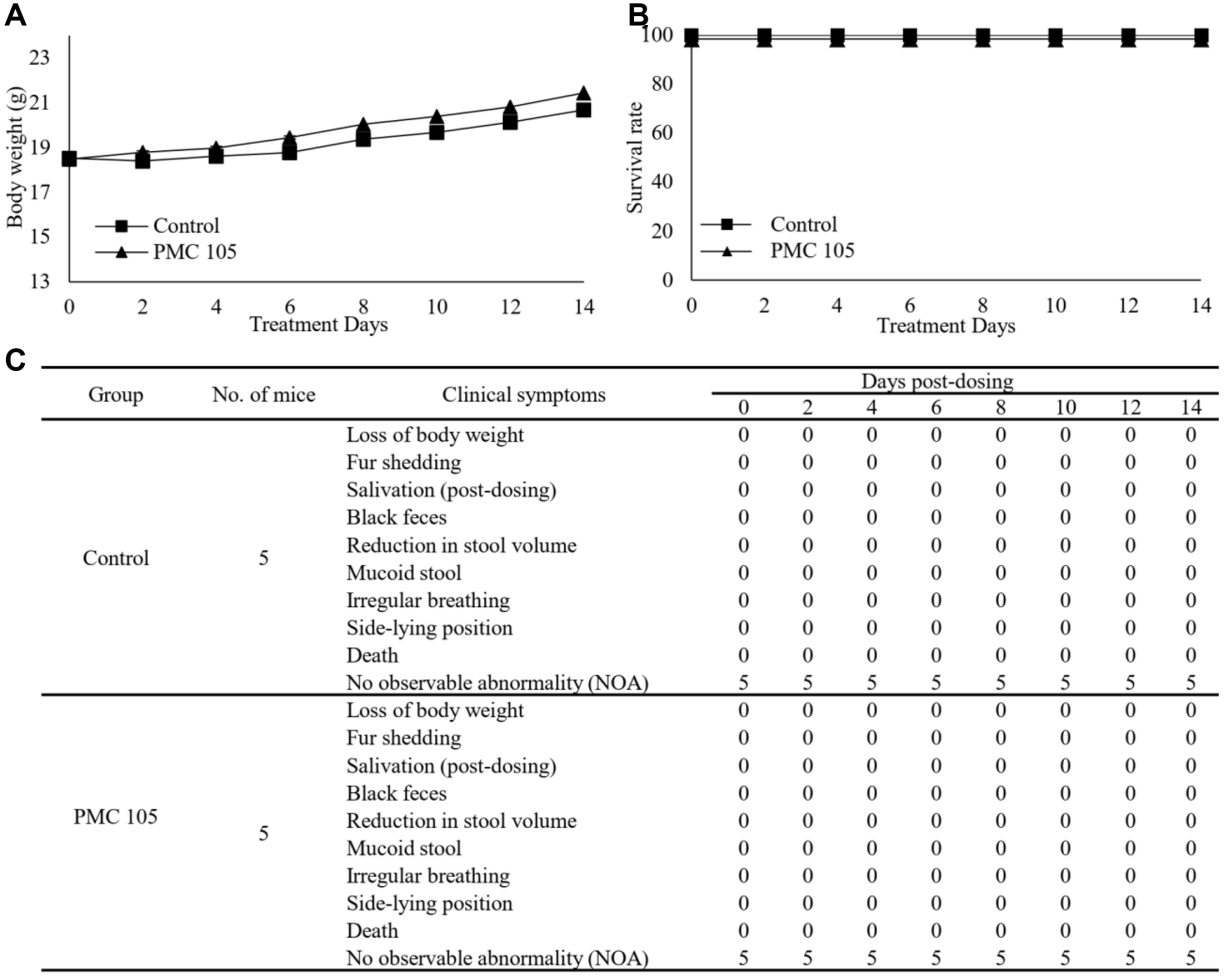
Oral acute toxicity examination of probiotic strain. Nine-week-old BALB/c mice in the treatment group received *L. plantarum* PMC105 via gavage (200 μl per mouse) twice a week, while the control group received only sterile water under the same conditions. (**A**) During the experimental period, the body weights of all mice were measured every 2 d for two weeks. There was no significant difference in body weight between PMC105-treated mice and untreated mice. (**B**) Mice’s survival was observed during the experiment. All mice in both groups survived till the end of the study. (**C**) Clinical symptoms and survival rates were evaluated in a mouse model of acute oral dose toxicity. No abnormalities were detected in the treated mice, similar to those in the untreated group. Each group consisted of five mice (*n* = 5). All data are presented as the mean ± SD.

An acute toxicity study of *L. plantarum* was conducted in a mouse model to assess potential side effects of the strain. Nine-week-old mice were administered *L. plantarum* at the maximum achievable dose levels (200 μl per mouse). Mortality, general appearance, and body weight were monitored for 14 d. No abnormal clinical signs or significant differences in body weight were observed between the mice treated with *L. plantarum* and those receiving sterile water. All mice survived throughout the experiment, and no adverse effects or mortality were observed in any group (Fig. 5).

### Comprehensive Safety Assessment of the Probiotic Candidate

**Fig. 6:**
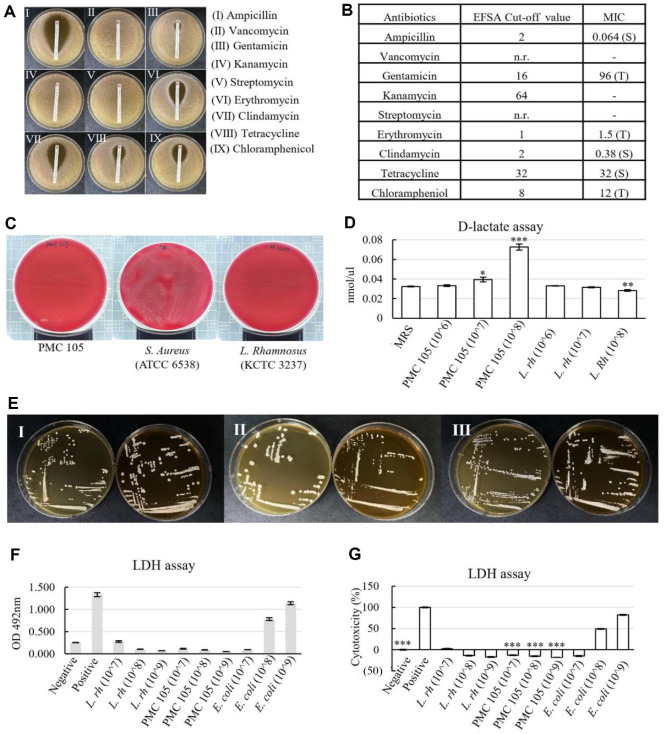
Safety Evaluation of Probiotic Strain. To ensure the safety of probiotic strains for therapeutic use, antibiotic susceptibility, hemolytic activity, D–lactic acid production, bile salt deconjugation activity, and cell cytotoxicity were examined. (**A**) E–test results for the determination of antibiotic resistance showed susceptibility to ampicillin, clindamycin, and tetracycline, with tolerance to gentamicin, erythromycin, and chloramphenicol observed. (**B**) MIC values were compared to EFSA standards to confirm acceptable resistance profiles (S, susceptible; T, tolerance; n.r., not required). (**C**) *L. plantarum* PMC105 demonstrated γ–hemolytic activity (absence of hemolysis) in the blood agar hemolysis assay, consistent with the probiotic control *L. rhamnosus* KCTC 3237, while *Staphylococcus aureus* ATCC 6538 exhibited β–hemolysis (positive control). (**D**) D–lactate production of *L. plantarum* PMC105 and *L. rhamnosus* KCTC3237 was measured in mmol/μl and exhibited detectable levels. (**E**) Deconjugation of bile salt assay showing bile salt hydrolase activity in *L. plantarum* PMC105 (Series I) along with negative and positive controls on plates with and without 0.5% TDCA. *L*. Series II; *L. rhamnosus* KCTC3237, series III; *L. plantarum* KCTC3105. (**F, G**) Cytotoxicity of PMC105 on HT−29 cells was assessed using the LDH release assay. No significant cytotoxic effects were detected compared to the negative control, while *E. coli* served as a positive control. All experiments were performed in triplicate. All data are expressed as the mean ± SD. Statistical significance was indicated as a *p*–value of less than 0.05; *p < 0.05, ** *p* < 0.01, ***p < 0.001.

Fig. 6A and 6B demonstrate the profiles of antibiotic susceptibility of the probiotic strain. The *L. plantarum* PMC105 was susceptible to clindamycin, tetracycline, and ampicillin, but tolerant to gentamicin, kanamycin, erythromycin, and chloramphenicol.

Bacterial infections, including invasive infections, can often lead to hemolytic symptoms, such as anemia, fever, and skin rashes [54]. Thus, investigating the hemolytic activity of probiotics is crucial to confirm their safety. The hemolytic activity of *L. plantarum* PMC105 was assessed using blood agar plates by monitoring the presence or absence of clear (β–hemolysis), greenish (α–hemolysis), or no zones (γ–hemolysis) around the bacterial colonies after incubation. In the current study, the probiotic strain did not produce any clear or greenish zones around its colonies, indicating γ–hemolytic activity (Fig. 6C). This demonstrates *L. plantarum* PMC105 did not lyse red blood cells (RBCs) in the examination, confirming its non-hemolytic nature.

Fig. 6D illustrates the D–lactate production results, with concentrations expressed in mmol/μl. *L. rhamnosus* KCTC3237 and *L. plantarum* PMC105 were evaluated using the D–lactate kit. When the CFU of *L. plantarum* PMC105 gradually increased, the level of D–lactate production increased. This observation is notable, as elevated D–lactate levels can cause D–lactic acidosis, posing potential safety concerns in probiotic applications. Conversely, the minimal or undetectable D–lactate production in the other CFUs indicates their suitability for probiotic use, minimizing the risk of D–lactic acidosis in clinical conditions. This characteristic is essential to ensure a favorable safety profile during drug development.

In this study, we investigated bile salt deconjugation activity using the standard plate assay method. In agar plates containing taurodeoxycholic acid (TDCA), one of the major conjugated bile salts in the human gastrointestinal tract, overall deconjugation was observed by strains PMC 105, as well as control strains *L. rhamnosus* KCTC3237 and *L. plantarum* KCTC3105 (Fig. 6E). The bile salt deconjugation activity is an important property of probiotics, as it promotes their survival in the gastrointestinal tract by improving bile salt tolerance, an essential factor to maintain their viability and functionality in the challenging intestinal environment.

Fig. 6F and 6G show the cytotoxicity of *L. plantarum* PMC105, as measured by lactate dehydrogenase (LDH). The results showed that *L. plantarum* PMC105 has no cytotoxic effect on the HT29 cell line.

## Discussion

The opportunistic pathogen CRKP, which is resistant to a wide range of antibiotics, poses a significant threat. The World Health Organization lists CRKP as a critical priority antibiotic-resistant pathogen for which new treatments are urgently needed [55]. Previous research has shown that by modulating the immune system and maintaining intestinal cell homeostasis, metabolites and functional factors from the gut microbiota strongly impact the host gastrointestinal tract [56]. Probiotics are promising candidates for treating multidrug-resistant infections.

*L. plantarum* is known as a potential antimicrobial probiotic against a broad range of gastrointestinal pathogens, such as *Es. coli* and *S. aureus*, with different mechanisms that include the production of bacteriocins, immune system modulation, production of short fatty acids, and maintenance of the intestinal barrier integrity [57]. In contrast, *Listeria monocytogenes* is known for its antibacterial metabolites [58]. In this study, *L. plantarum* PMC105, isolated from a traditional Korean fermented kimchi, was evaluated for its potential to prevent or suppress CRKP infection, focusing on its safety profile, effects on intestinal barrier function, and clinical outcomes in mouse models.

Several studies have proven the tolerance of *L. plantarum* in gastrointestinal conditions. *L. plantarum* is resistant to low pH conditions and can ferment lactic and deconjugate bile salts. Lactic acid fermentation helps *L. plantarum* survive in a gut-acidic environment while reducing the risk of metabolic acidosis, thereby improving gut health [59].

In this study, the oral preventive and intranasal nebulization models were utilized to evaluate the therapeutic efficacy of *L. plantarum* PMC105 against CRKP infection in immunocompromised mice. The preventive model, which involved oral administration of *L. plantarum* prior to CRKP infection, aimed to inhibit initial colonization and improve gut barrier function. In contrast, the nebulization model assessed the strain’s therapeutic potential post-infection, targeting reductions in systemic bacterial burden and illness severity. The preventive model showed strong protective potential, with treated mice showing significantly reduced illness severity, decreased CRKP colonization in the gut, improved maintenance of body weight, and 100% survival, compared to a 60%mortality rate in the untreated group. In addition, PMC105 maintained intestinal barrier integrity, as indicated by preserved ZO−1 protein expression levels in Western blot assays. This indicates that *L. plantarum* improves the intestinal barrier function and supports the local immune response prior to infection. Although the nebulization model was applied after CRKP infection, it still demonstrated promising outcomes. The intranasal delivery of *L. plantarum* resulted in reduced bacterial loads in stool and organs, enhanced clinical condition, and improved survival rate. These findings indicate that even after pathogen colonization, *L. plantarum* may provide systemic protective effects, potentially by modulating mucosal immune responses or inhibiting bacterial spread. Comparison between the two models showed that oral treatment achieved a higher survival rate, suggesting a more effective role in blocking CRKP colonization and infection progression [60, 61].

Statistical analysis of the oral preventive mouse model showed that *L. plantarum* decreased the average illness severity score by ~66.7%, and increased body weight by approximately 230% by D 16, compared to the untreated group. All treated mice survived in the preventive model study; however, the nebulization-treated group demonstrated 91.66% survival compared with untreated mice by 58.33% of survival rate. Furthermore, to compare with a previous study using *Bacillus velezensis*, which reported that 80% of mice survived, *L. plantarum* PMC105 demonstrated greater preventive efficacy against CRKP infection [33].

Our findings also support the anti-colonization potential of *L. plantarum*. While *L. fermentum* has been shown to reduce CRKP intestinal colonization by approximately 27% [33], *L. plantarum* in our study achieved a 50%reduction, along with a ~ 66.7% decrease in illness severity, and a decrease in weight loss by about 39%. These outcomes emphasize that, compared to *L. fermentum*, *L. plantarum* is more effective at suppressing CRKP colonization in the gastrointestinal tract, making it an eligible candidate for suppressing CRKP infection.

According to previous studies, the intestinal barrier plays a crucial role in maintaining gastrointestinal tract health by inhibiting the translocation of pathogens into the bloodstream. ZO−1, as the tight junction protein, is responsible for the maintenance of intestinal barrier integrity [62]. *L. plantarum* exerts its microbial activity by up-regulation of the tight junction protein ZO−1 gene expression, and significantly promotes the production of ZO−1 protein [63]. In this study, Western blot was used to analyze ZO−1 expression in mouse models. Our results demonstrated that *L. plantarum* treatment preserved ZO−1 expression, indicating its preventive effect on CRKP-induced intestinal damage and systemic spread.

Safety evaluation in alignment with international standards, including FAO/WHO and Korean MFDS guidelines [45, 46], confirmed that *L. plantarum* PMC105 is a non-hemolytic strain, supporting its non-pathogenic nature. The evaluation of toxin production, conducted using *in vitro* assays, showed no detectable toxin, confirming the strain’s safety. The strain did not produce excessive D–lactate, thereby minimizing the risk of metabolic acidosis, and it also showed low pH tolerance. In addition, its bile salt deconjugation activity indicates its survival in the gastrointestinal environment and its beneficial role in supporting healthy gut microbiota. Moreover, Antibiotic susceptibility testing revealed acceptable resistance profiles in accordance with EFSA guidelines [48]. PMC105 strain demonstrated susceptibility to a wide range of antibiotics, with no transferable resistance genes detected. Other previous studies have also reported the safety profile of *L. plantarum*, as it is susceptible to Tetracycline, Ampicillin, Vancomycin, Ciprofloxacin, Gentamicin, Azithromycin, and Kanamycin. Also, these studies showed that *L. plantarum* lacked hemolytic or *in vivo* toxicological activity [64]. Numerous *Lactobacilli* are resistant to aminoglycoside antibiotics, such as kanamycin [65, 66], likely due to reduced aminoglycoside uptake in the absence of cytochrome-mediated transport [67, 68]. For example, kanamycin resistance was observed in 79% of 187 isolates from 55 probiotic products in Europe [69]. This observation aligns with EFSA criteria, which indicate that aminoglycoside resistance in *Lactobacilli* is an intrinsic, non-transferable trait. Moreover, E-test susceptibility testing confirmed that *L. plantarum* PMC105 meets the safety standards for pharmaceutical applications.

In summary, this study demonstrated the effects of *L. plantarum* PMC105 on CRKP infection *in vivo* and *in vitro* and suggested that it could be a candidate for treating CRKP. This indicates it can be used as a biotherapeutic agent and in combination therapy. However, further studies are needed that focus more on the biological mechanisms underlying the beneficial effects of *L. plantarum*, including its interactions with the immune system, its impact on the gut microbiome, optimal doses, and possible therapeutic side effects. In addition, clinical trials will be essential to evaluate the efficacy and safety of *L. plantarum* in individuals affected by CRKP infections.

## Conclusion

**Fig. 7:**
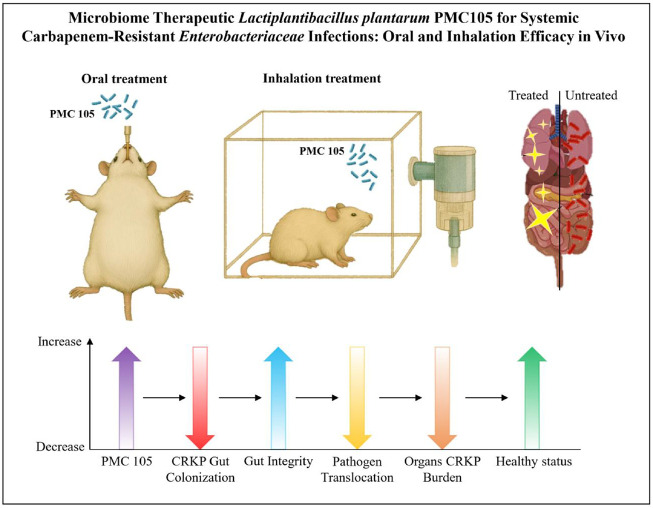
*L. plantarum* PMC105, delivered either orally before infection or by inhalation with nebulizer after infection, reduces CRKP colonization and systemic bacterial burden, improves intestinal barrier integrity, and supports organ health, highlighting its potential as a biotherapeutic strategy against multidrug-resistant infections.

This research reveals the promising therapeutic advantage of L. plantarum PMC105 in combating CRKP infections. Further, this study provides strong evidence through both preventive and post-infection murine models, supporting the effect of L. plantarum PMC105 as a promising probiotic to prevent and suppress CRKP infections. Its safety profile, ability to maintain intestinal barrier integrity, and efficacy in reducing illness severity make it a promising candidate for further research and possible clinical applications. Importantly, both oral and intranasal delivery of PMC105 resulted in protective benefits, demonstrating flexibility in its administration route for therapeutic purposes. Fig. 7 summarizes these findings, illustrating the reduction in CRKP burden and preservation of organ health following PMC105 treatment. Future work should focus on mechanistic pathways, optimal dosing, and clinical translation to evaluate PMC105 as a scalable probiotic therapeutic candidate.

## Figures and Tables

**Table 1:** Identification of the isolated bacterial strain based on 16S rRNA gene sequencing.

NCBI reference	Organism	Length	Score	Identities	Gaps
NR_115605.1	*Lactiplantibacillus plantarum* DSM 20174	1,519	2,713 bits (1,469)	1,470/1,471 (99 %)	0/1,471 (0 %)
NR_113338.1	*Lactiplantibacillus plantarum* NBRC 15891	1,492	2,713 bits (1,469)	1,470/1,471 (99 %)	0/1,471 (0 %)
NR_029133.1	*Lactiplantibacillus pentosus* 124-2	1,519	2,712 bits (1,468)	1,470/1,471 (99 %)	0/1,471 (0 %)
NR_025447.1	*Lactiplantibacillus paraplantarum* DSM 10667	1,502	2,699 bits (1,461)	1,469/1,473 (99 %)	0/1,473 (0 %)
NR_104573.1	*Lactiplantibacillus plantarum* CIP 103151	1,527	2,699 bits (1,461)	1,463/1,464 (99 %)	0/1,464 (0 %)
NR_042394.1	*Lactiplantibacillus plantarum* NRRL B-14768	1,474	2,699 bits (1,461)	1,463/1,464 (99 %)	0/1,464 (0 %)
NR_117813.1	*Lactiplantibacillus plantarum* DSM 20174	1,466	2,680 bits (1,451)	1,456/1,458 (99 %)	1/1,458 (0 %)
NR_042254.1	*Lactiplantibacillus argentoratensis* DKO 22	1,517	2,662 bits (1,441)	1,458/1,469 (99 %)	2/1,469 (0 %)
NR_112690.1	*Lactiplantibacillus plantarum* NBRC 15891	1,454	2,643 bits (1,431)	1,432/1,433 (99 %)	0/1,433 (0 %)
NR_042676.1	*Lactiplantibacillus fabifermentans* LMG 24284	1,532	2,632 bits (1,425)	1,458/1,474 (99 %)	1/1,474 (0 %)
NR_113339.1	*Lactiplantibacillus fabifermentans* DSM 21115	1,491	2,627 bits (1,422)	1,455/1,471 (99 %)	1/1,471 (0 %)
NR_136785.1	*Lactiplantibacillus plajomi* NB53	1,492	2,623 bits (1,420)	1,454/1,471 (99 %)	0/1,471 (0 %)
NR_136786.1	*Lactiplantibacillus modestisalitolerans* NB446	1,492	2,603 bits (1,409)	1,450/1,471 (99 %)	0/1,471 (0 %)

**Table 2:** Comparison of the chromosomal properties of *Lactiplantibacillus plantarum* strains.

Strain	PMC105	LPJBC5	B4	LPgoji	KU210152	L75	BIA
Source	kimchi	curd	blueberry	wolfberry	kimchi	Elymus nutan	orange
Genome size (bp)	3,276,265	3,231,898	3,217,780	3,234,760	3,206,954	3,198,110	3,238,827
G+C content (%)	44.5	44.5	44.5	44.5	44	44.5	44.5
Predicted CDS	3,054	2,906	2,923	2,844	3,157	3,091	2,979
Number of rRNA genes	16	16	16	16	21	16	16
Number of tRNA genes	74	67	72	71	87	69	73

LPGBC5: https://www.ncbi.nlm.nih.gov/nuccore/NZ_CP185854.1

B4: https://www.ncbi.nlm.nih.gov/nuccore/CP096862.1

LPgogi: https://www.ncbi.nlm.nih.gov/nuccore/CP096853.1

KU210152: https://www.ncbi.nlm.nih.gov/nuccore/NZ_CP187835.1

L75: https://www.ncbi.nlm.nih.gov/nuccore/NZ_CP151092.1

BIA: https://www.ncbi.nlm.nih.gov/nuccore/CP170649.1

NC: not confirmed.

## References

[1] Lei TY, Liao BB, Yang LR, Wang Y, Chen XB (2024). Hypervirulent and carbapenem-resistant *Klebsiella pneumoniae*: a global public health threat. Microbiol. Res..

[2] Chang PC, Chen CC, Lu YC, Lai CC, Huang HL, Chuang YC (2018). The impact of inoculum size on the activity of cefoperazonesulbactam against multidrug resistant organisms. J. Microbiol. Immunol. Infect..

[3] Choby JE, Howard-Anderson J, Weiss DS (2020). Hypervirulent *Klebsiella pneumoniae* - clinical and molecular perspectives. J. Intern. Med..

[4] Abbas R, Chakkour M, Zein El Dine H, Obaseki EF, Obeid ST, Jezzini A (2024). General overview of *Klebsiella pneumonia*: epidemiology and the role of siderophores in its pathogenicity. Biology (Basel).

[5] Vazquez-Ucha JC, Arca-Suarez J, Bou G, Beceiro A (2020). New carbapenemase inhibitors: clearing the way for the beta-lactams. Int. J. Mol. Sci..

[6] Abdel-Halim MS, Askoura M, Mansour B, Yahya G, El-Ganiny AM (2022). *In vitro* activity of celastrol in combination with thymol against carbapenem-resistant *Klebsiella pneumoniae* isolates. J. Antibiot (Tokyo).

[7] Do AD, Quang HP, Phan QK (2025). Probiotic cell-free supernatant as effective antimicrobials against *Klebsiella pneumoniae* and reduce antibiotic resistance development. Int. Microbiol..

[8] Hill C, Guarner F, Reid G, Gibson GR, Merenstein DJ, Pot B (2014). Expert consensus document. The international scientific association for probiotics and prebiotics consensus statement on the scope and appropriate use of the term probiotic. Nat. Rev. Gastroenterol. Hepatol..

[9] Yousefi B, Eslami M, Ghasemian A, Kokhaei P, Salek Farrokhi A, Darabi N (2019). Probiotics importance and their immunomodulatory properties. J. Cell. Physiol..

[10] Squarzanti DF, Dell'Atti F, Scalia AC, Najmi Z, Cochis A, Malfa P (2024). Exploring the *in vitro* antibacterial potential of specific probiotic strains against oral pathogens. Microorganisms.

[11] Zommiti M, Chikindas ML, Ferchichi M (2020). Probiotics-live biotherapeutics: a story of success, limitations, and future prospectsnot only for humans. Probiotics Antimicrob. Proteins.

[12] Jin Y, Lu Y, Jiang X, Wang M, Yuan Y, Zeng Y (2024). Accelerated infected wound healing by probiotic-based living microneedles with long-acting antibacterial effect. Bioact. Mater..

[13] Chen CC, Lai CC, Huang HL, Su YT, Chiu YH, Toh HS (2021). Antimicrobial ability and mechanism analysis of *Lactobacillus* species against carbapenemase-producing Enterobacteriaceae. J. Microbiol. Immunol. Infect..

[14] Tajdozian H, Seo H, Jeong Y, Ghorbanian F, Park C-e, Sarafraz F, *et al*. 2024. Efficacy of lyophilized *Lactobacillus sakei* as a potential candidate for preventing carbapenem-resistant *Klebsiella* infection. *Ann. Microbiol.* **74.** https://doi.org/10.1186/s13213-024-01773-8. 10.1186/s13213-024-01773-8

[15] Chen CC, Lai CC, Huang HL, Huang WY, Toh HS, Weng TC (2019). Antimicrobial activity of *Lactobacillus* species against carbapenem-resistant enterobacteriaceae. Front. Microbiol..

[16] Tang HJ, Chen CC, Lu YC, Huang HL, Chen HJ, Chuang YC (2022). The effect of *Lactobacillus* with prebiotics on KPC-2-producing *Klebsiella pneumoniae*. Front. Microbiol..

[17] Zhang C, Wang C, Dai J, Xiu Z (2024). The inhibition mechanism of co-cultured probiotics on biofilm formation of *Klebsiella pneumoniae*. J. Appl. Microbiol..

[18] Li J, Yu J, Song Y, Wang S, Mu G, Tuo Y (2024). Exopolysaccharides and surface-layer proteins expressed by biofilm-state *Lactiplantibacillus plantarum* Y42 play crucial role in preventing intestinal barrier and immunity dysfunction of Balb/C mice infected by *Listeria monocytogenes* ATCC 19115. J. Agric. Food Chem..

[19] Zhang S, Liu T, Zhou X, Wang J, Zhang T, Xiao G (2024). Isolation of *Lactiplantibacillus plantarum* for treatment of Salmonella infection in mice. Lett. Appl. Microbiol..

[20] Li H, Ma X, Shang Z, Liu X, Qiao J (2024). *Lactobacillus acidophilus* alleviate *Salmonella* enterica *Serovar* Typhimurium-induced murine inflammatory/oxidative responses via the p62-Keap1-Nrf2 signaling pathway and cecal microbiota. Front. Microbiol..

[21] Kumar M, Dhaka P, Vijay D, Vergis J, Mohan V, Kumar A (2016). Antimicrobial effects of *Lactobacillus plantarum* and *Lactobacillus acidophilus* against multidrug-resistant enteroaggregative *Escherichia coli*. Int. J. Antimicrob. Agents.

[22] Gopal PK, Prasad J, Smart J, Gill HS (2001). *In vitro* adherence properties of *Lactobacillus rhamnosus* DR20 and *Bifidobacterium lactis* DR10 strains and their antagonistic activity against an enterotoxigenic *Escherichia coli*. Int. J. Food Microbiol..

[23] Karska-Wysocki B, Bazo M, Smoragiewicz W (2010). Antibacterial activity of *Lactobacillus acidophilus* and *Lactobacillus casei* against methicillin-resistant *Staphylococcus aureus* (MRSA). Microbiol. Res..

[24] Mirnejad R, Vahdati AR, Rashidiani J, Erfani M, Piranfar V (2013). The antimicrobial effect of *Lactobacillus casei* culture supernatant against multiple drug resistant clinical isolates of *Shigella sonnei* and *Shigella flexneri*
*in vitro*. Iran. Red Crescent Med. J..

[25] McFarland LV. 2015. Probiotics for the primary and secondary prevention of *C. difficile* infections: a meta-analysis and systematic review. *Antibiotics (Basel)* **4:** 160-178. 10.3390/antibiotics4020160 27025619 PMC4790329

[26] Pompilio A, Kaya E, Lupetti V, Catelli E, Bianchi M, Maisetta G (2024). Cell-free supernatants from *Lactobacillus* strains exert antibacterial, antibiofilm, and antivirulence activity against Pseudomonas aeruginosa from cystic fibrosis patients. Microbes Infect..

[27] Mahdi LH, Jabbar HS, Auda IG (2019). Antibacterial immunomodulatory and antibiofilm triple effect of Salivaricin LHM against *Pseudomonas aeruginosa* urinary tract infection model. Int. J. Biol. Macromol..

[28] Makras L, Triantafyllou V, Fayol-Messaoudi D, Adriany T, Zoumpopoulou G, Tsakalidou E (2006). Kinetic analysis of the antibacterial activity of probiotic lactobacilli towards *Salmonella enterica* serovar Typhimurium reveals a role for lactic acid and other inhibitory compounds. Res. Microbiol..

[29] Spacova I, Petrova MI, Fremau A, Pollaris L, Vanoirbeek J, Ceuppens JL (2019). Intranasal administration of probiotic *Lactobacillus rhamnosus* GG prevents birch pollen-induced allergic asthma in a murine model. Allergy.

[30] Harata G, He F, Hiruta N, Kawase M, Kubota A, Hiramatsu M (2010). Intranasal administration of *Lactobacillus rhamnosus* GG protects mice from H1N1 influenza virus infection by regulating respiratory immune responses. Lett. Appl. Microbiol..

[31] Chen CM, Yang YSH, Chou HC, Lin S (2023). Intranasal administration of *Lactobacillus johnsonii* attenuates hyperoxia-induced lung injury by modulating gut microbiota in neonatal mice. J. Biomed. Sci..

[32] Hori T, Kiyoshima J, Shida K, Yasui H (2001). Effect of intranasal administration of *Lactobacillus casei* Shirota on influenza virus infection of upper respiratory tract in mice. Clin. Diagn. Lab. Immunol..

[33] Tajdozian H, Seo H, Kim S, Rahim MA, Lee S, Song HY (2021). Efficacy of *Lactobacillus fermentum* isolated from the vagina of a healthy woman against carbapenem-resistant *Klebsiella* infections *in vivo*. J. Microbiol. Biotechnol..

[34] Liu W, Bao Q, Jirimutu, Qing M, Siriguleng, Chen X (2012). Isolation and identification of lactic acid bacteria from Tarag in Eastern Inner Mongolia of China by 16S rRNA sequences and DGGE analysis. Microbiol. Res..

[35] Hyatt D, Chen GL, Locascio PF, Land ML, Larimer FW, Hauser LJ (2010). Prodigal: prokaryotic gene recognition and translation initiation site identification. BMC Bioinformatics.

[36] Lowe TM, Eddy SR (1997). tRNAscan-SE: a program for improved detection of transfer RNA genes in genomic sequence. Nucleic Acids Res..

[37] Nawrocki EP, Burge SW, Bateman A, Daub J, Eberhardt RY, Eddy SR, *et al*. 2015. Rfam 12.0: updates to the RNA families database. *Nucleic Acids Res*. **43:** D130-137. 10.1093/nar/gku1063 25392425 PMC4383904

[38] Yoon SH, Ha SM, Lim J, Kwon S, Chun J (2017). A large-scale evaluation of algorithms to calculate average nucleotide identity. Antonie Van Leeuwenhoek.

[39] Abdelhalim MM, Saafan GS, El-Sayed HS, Ghaith DM (2022). *In vitro* antibacterial effect of probiotics against Carbapenamaseproducing multidrug-resistant *Klebsiella pneumoniae* clinical isolates, Cairo, Egypt. J. Egypt Public Health Assoc..

[40] Soundharrajan I, Yoon YH, Muthusamy K, Jung JS, Lee HJ, Han OK (2021). Isolation of *Lactococcus lactis* from whole crop rice and determining its probiotic and antimicrobial properties towards gastrointestinal associated bacteria. Microorganisms.

[41] Soundharrajan I, Kim D, Kuppusamy P, Muthusamy K, Lee HJ, Choi KC (2019). Probiotic and triticale silage fermentation potential of *Pediococcus pentosaceus* and *Lactobacillus brevis* and their impacts on pathogenic bacteria. Microorganisms.

[42] Rahim MA, Seo H, Kim S, Barman I, Ghorbanian F, Hossain MS (2024). Exploring the potential of *Lactocaseibacillus rhamnosus* PMC203 in inducing autophagy to reduce the burden of *Mycobacterium tuberculosis*. Med. Microbiol. Immunol..

[43] Peng Z, Bao L, Shi B, Shi YB (2024). Protein arginine methyltransferase 1 is required for the maintenance of adult small intestinal and colonic epithelial cell homeostasis. Int. J. Biol. Sci..

[44] Thumu SCR, Halami PM (2020). *In vivo* safety assessment of *Lactobacillus fermentum* strains, evaluation of their cholesterol-lowering ability and intestinal microbial modulation. J. Sci. Food Agric..

[45] 2002. FAO. FAO/WHO. Guidelines for the Evaluation of Probiotics in Food. Available at https://isappscience.org/wp-content/uploads/2019/04/probiotic_guidelines.pdf [accessed on 4 October 2024].

[46] 2021. KMFDS. (2021). Guidelines for the safety evaluation of probiotics as functional ingredients in health functional foods. .

[47] Nachnani S, Scuteri A, Newman MG, Avanessian AB, Lomeli SL (1992). E-test: a new technique for antimicrobial susceptibility testing for periodontal microorganisms. J. Periodontol..

[48] Zhou JS, Pillidge CJ, Gopal PK, Gill HS (2005). Antibiotic susceptibility profiles of new probiotic *Lactobacillus* and *Bifidobacterium* strains. Int. J. Food Microbiol..

[49] 2012. Guidance on the assessment of bacterial susceptibility to antimicrobials of human and veterinary importance. *EFSA J.* **10.**

[50] Halder D, Mandal M, Chatterjee SS, Pal NK, Mandal S (2017). Indigenous probiotic *Lactobacillus* isolates presenting antibiotic like activity against human pathogenic bacteria. Biomedicines.

[51] Ahn YT, Kim GB, Lim KS, Baek YJ, Kim HU (2003). Deconjugation of bile salts by *Lactobacillus* acidophilus isolates. International Dairy Journal..

[52] Goffin P, Deghorain M, Mainardi JL, Tytgat I, Champomier-Verges MC, Kleerebezem M (2005). Lactate racemization as a rescue pathway for supplying D-lactate to the cell wall biosynthesis machinery in *Lactobacillus* plantarum. J. Bacteriol..

[53] 2021. Guidelines for the safety evaluation of probiotics as functional ingredients in health functional foods. 2021.

[54] Bang WY, Chae SA, Ban OH, Oh S, Park C, Lee M (2021). The *in vitro* and *in vivo* safety evaluation of *Lactobacillus acidophilus* IDCC 3302. Microbiol. Biotechnol. Lett..

[55] David S, Reuter S, Harris SR, Glasner C, Feltwell T, Argimon S (2019). Epidemic of carbapenem-resistant *Klebsiella pneumoniae* in Europe is driven by nosocomial spread. Nat. Microbiol..

[56] Yan F, Polk DB (2020). Probiotics and probiotic-derived functional factors-mechanistic insights into applications for intestinal homeostasis. Front. Immunol..

[57] Xu Z, Wang J, Zheng W, Zhao S, Yang Q, Yu D (2022). Antibacterial activity and mechanism of a novel bacteriocin produced by *Lactiplantibacillus plantarum* against *Escherichia coli* and *Staphylococcus aureus*. Int. J. Food Sci. Technol..

[58] Li Y, Yu S, Weng P, Wu Z, Liu Y. 2023. Purification and antimicrobial mechanism of a novel bacteriocin produced by *Lactiplantibacillus plantarum* FB-2. *LWT* **185.** 10.1016/j.lwt.2023.115123

[59] Echegaray N, Yilmaz B, Sharma H, Kumar M, Pateiro M, Ozogul F (2023). A novel approach to *Lactiplantibacillus plantarum*: From probiotic properties to the omics insights. Microbiol. Res..

[60] Zhang M, Qin Z, Huang C, Liang B, Zhang X, Sun W (2025). The gut microbiota modulates airway inflammation in allergic asthma through the gut-lung axis related immune modulation: A review. Biomol. Biomed..

[61] Meijerink M, Wells JM (2010). Probiotic modulation of dendritic cells and T cell responses in the intestine. Benef. Microbes.

[62] Vancamelbeke M, Vermeire S (2017). The intestinal barrier: a fundamental role in health and disease. Expert Rev. Gastroenterol. Hepatol..

[63] Bu Y, Liu Y, Liu Y, Cao J, Zhang Z, Yi H (2023). Protective effects of bacteriocin-producing *Lactiplantibacillus plantarum* on intestinal barrier of mice. Nutrients.

[64] Ghorbanian F, Seo H, Tajdozian H, Lee Y, Rahim MA, Kim S (2022). *In vivo* efficacy of *Bacillus velezensis* isolated from Korean Gochang bokbunja vinegar against carbapenem-resistant *Klebsiella pneumoniae* infections. Pol. J. Microbiol..

[65] Ammor MS, Florez AB, Mayo B (2007). Antibiotic resistance in non-enterococcal lactic acid bacteria and bifidobacteria. Food Microbiol..

[66] Abriouel H, Casado Munoz MDC, Lavilla Lerma L, Perez Montoro B, Bockelmann W, Pichner R (2015). New insights in antibiotic resistance of *Lactobacillus* species from fermented foods. Food Res. Int..

[67] Mayrhofer S, van Hoek AH, Mair C, Huys G, Aarts HJ, Kneifel W (2010). Antibiotic susceptibility of members of the *Lactobacillus acidophilus* group using broth microdilution and molecular identification of their resistance determinants. Int. J. Food Microbiol..

[68] Charteris WP, Kelly PM, Morelli L, Collins JK (2001). Gradient diffusion antibiotic susceptibility testing of potentially probiotic lactobacilli. J. Food Prot..

[69] Temmerman R, Pot B, Huys G, Swings J (2003). Identification and antibiotic susceptibility of bacterial isolates from probiotic products. Int. J. Food Microbiol..

